# Occurrence and concentration of caffeine and cadmium as micropollutants in the Red Sea coast, Egypt

**DOI:** 10.1038/s41598-026-38344-7

**Published:** 2026-02-23

**Authors:** Samaa G. El-Sokkary, Khaleid F. Abd El-Wakeil, Ahmad H. Obuid-Allah

**Affiliations:** Zoology and Entomology Department, Faculty of Science, Assiut University, Assiut, Egypt

**Keywords:** Pharmaceutical residues, Heavy metals, Anthropogenic impacts, Physicochemical variables, Coastal ecosystem, Ecology, Ecology, Environmental sciences

## Abstract

This study investigated the occurrence and levels of caffeine residue and cadmium traces on the Red Sea coast of Egypt and the effects of environmental variables on caffeine concentration, in order to evaluate the anthropogenic impacts on the coastal area of the Red Sea. To accomplish this study; three different sites were taken: El-Hamraween (HMR), Abo El-Swater (SWT), and Om El-Abas (ABS). Sediment and water samples were collected from low and high intertidal zones during June 2023. Some physicochemical variables and concentrations of caffeine and Cd were measured in the collected samples. Caffeine concentration in water samples ranged from 10.94 to 14.17 µg/L at low intertidal zone in SWT and the high intertidal zone in ABS, respectively, while caffeine concentration in sediment ranged from 0.27 to 0.66 µg/g at low intertidal zone in ABS and the high intertidal zone in HMR, respectively. The results indicate significant differences among the investigated sites according to variations in anthropogenic impacts. This study showed a clear association between caffeine and cadmium concentrations with physicochemical variables. Caffeine and Cd’s correlation and anthropogenic impacts highlight the need for more research on the interactions between pharmaceutical residues and heavy metals and their effects on the environment.

**Supplementary Information:**

The online version contains supplementary material available at 10.1038/s41598-026-38344-7.

## Introduction

Seawater covers 97% of all surface waters and is considered one of the most abundant resources on our planet^1^. Human activities have led to the unsustainable use of marine waters and resources, resulting in altered marine ecosystems, a change linked to eutrophication, loss of biodiversity, or the presence of pollutants^2^. The Red Sea is a semi-enclosed tropical marine body located between the Mediterranean Sea and the Indian Ocean^3^. The water quality of the Red Sea has become an increasingly important subject of investigation because of its ecological significance and economic implications. This unique marine environment hosts one of the most biologically diverse ecosystems on Earth, contributing extensive biodiversity^4^. Historically, the Red Sea has been perceived as relatively free from pollution^5^. However, the accelerated development of coastal areas along the Egyptian Red Sea is now posing significant environmental risks stemming from a variety of human activities like fisheries production, phosphate in ports, and the tourism industry^6^.

Numerous emerging contaminants, particularly pharmaceutical and personal care products (PPCPs), are increasingly threatening aquatic ecosystems because of their widespread and largely unregulated use in human and veterinary medicine^7^. The residues of these substances are now commonly found in surface water, groundwater, oceans, and even drinking water, often entering the environment through public effluents and inadequately treated wastewater^8^. Despite growing global concern, conventional wastewater treatment plants are largely ineffective at removing these compounds, leading to their persistent discharge and accumulation^9^. Pharmaceutical residues pose serious environmental risks, contaminating soils and water bodies, impacting non-target organisms, and potentially transferring through food chains to humans and animals^10–13^.The possible marine effects of pharmaceutical discharges from sewage and other sources onto coastal habitats have received far less consideration^14^. Waste management from coastal megacities is becoming more widely acknowledged as a significant concern, and global demographic trends toward coastal conurbations indicate that more people live near coasts^15,16^. Generally, if the discharge of pharmaceuticals into coastal ecosystems reaches levels that cause biological effects, these substances could serve as extra stressors on marine environments that are already affected by climate change, eutrophication, and overfishing^17^.

Caffeine, a widely consumed central nervous system stimulant found in various foods, beverages, and medications, is one of the most prevalent pharmaceutical residues detected in aquatic environments^9^. The amount of caffeine that enters the water is significantly higher than that which is broken down, even though caffeine has exceptional removal efficiency during wastewater treatment^18^. Detection of caffeine in remote regions like Antarctica, highlighting its global reach and persistence^19^. Caffeine’s consistent detection worldwide has led to its classification as an emerging pollutant and a reliable indicator of anthropogenic and wastewater contamination^20^. According to an analysis of the literature by Vieira et al.^21^, caffeine concentrations have been found in coastal ecosystems, which has raised serious concerns about possible negative effects on human health and ecological safety. They demonstrated that caffeine has been detected in tissues from marine and coastal biota as a result of bioaccumulation following prolonged, chronic exposure to contaminated environments. As a result, they emphasize how critical it is to reduce the rising amount of caffeine that enters aquatic ecosystems. As such, scientific programs and projects must be put in place to properly classify caffeine as a high-priority environmentally hazardous emerging pollutant. Caffeine has emerged as a significant environmental contaminant, with numerous hotspots of contamination identified globally within marine ecosystems^10,16,22^.

Heavy metals are intrinsic constituents of the Earth’s crust and typically enter aquatic systems through natural processes such as the weathering of geological formations, soil erosion, and volcanic eruptions. However, the main route of heavy metal introduction into aquatic environments is through anthropogenic sources, including mining, maritime activities, and discharges from industrial, municipal, and agricultural operations^23,24^. In aquatic ecosystems, sediments are the primary sink for these metals, serving both as a reservoir and a potential source of bioavailable metals that can be taken up by aquatic organisms^25^. Paul et al.^26^ demonstrated that the majority of research on trace metals in coastal sediments concentrates on lead (Pb), zinc (Zn), and cadmium (Cd) because of their toxicity to humans and marine life as well as their strong and widespread enrichments as a result of extensive (pre)industrial use.

Cadmium (Cd) is generally classified as a toxic trace element. Many previous studies have recorded considerably high Cd concentrations along the Egyptian Red Sea coast^6,27–34^. El-Metwally et al.^6,28^ illustrated that cadmium has a higher enrichment factor (EF) value in Red Sea sediments indicated strongly contamination condition. Mohamed et al.^32^ mentioned that over 50 years the environmental studies focused on the pollution of coastal habitats in the Red Sea and Gulf of Suez. They showed that Cd exceeded the threshold for the WHO guidelines and exhibited a comparatively higher level of danger due to industrial pollution. Mohammed et al.^33^ examined the presence of heavy metals in mangrove sediment at 22 sites along the Egyptian Red Sea coast. They found that the degree of Cd contamination was considerably high (8 sites) to very high (14 sites) in all studied sites. They explained that Cd accumulation on the Egyptian Red Sea sediment originated from anthropogenic sources^35^ and natural sources from Red Sea hills, as recorded by Hanna^36^. According to Hanna^36^, the combined cadmium content of Red Sea sediment samples collected during the 1934 and 1984 expeditions ranged between 0.1 and 2 mg/kg, suggesting that its source is linked to lithogenous marine sediments.

Tawfik et al.^9^ emphasized the importance study of interactions between heavy metals and pharmaceutical residues as well as their bioavailability as a joint concern to the environment and human health. In this sense, the present study is the first combined assessment of caffeine and Cd in the Red Sea. The primary aim of was to provide a preliminary detection of caffeine concentrations on the Red Sea coast of Egypt and examine its correlation with cadmium and some physicochemical variables (water temperature, pH, conductivity, dissolved oxygen, total dissolved solids, and sediment organic matter and carbonate content and sediment grains size). Also, the study aimed to discuss the anthropogenic impacts on the concentrations of the two investigated micropollutants, caffeine and cadmium.

## Materials and methods

### Sampling

**Fig. 1:**
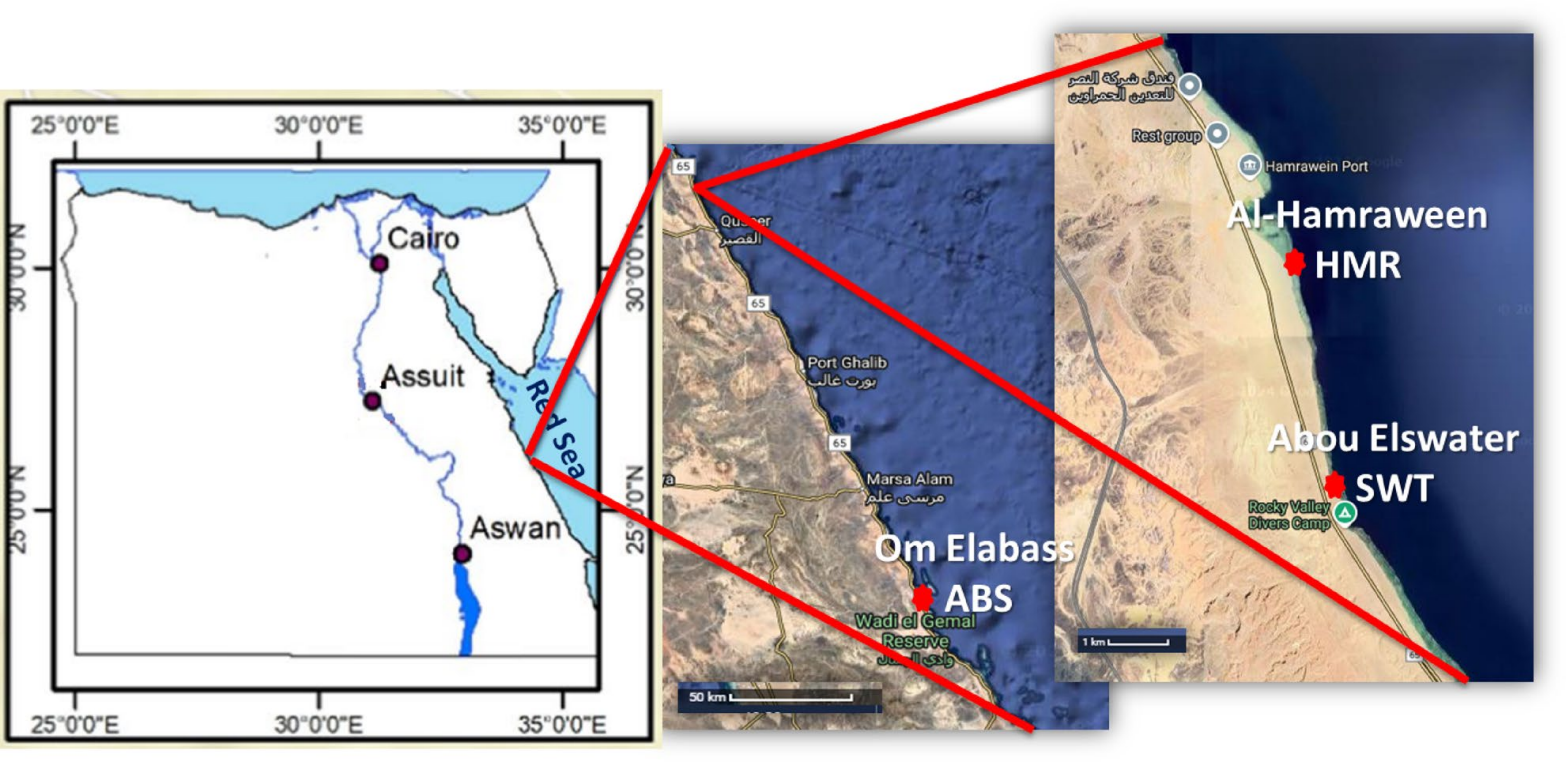
Maps show the investigated sites on Red Sea Coast in Egypt. El-Hamraween (HMR), Abo El-Swater (SWT) and Om El-Abas (ABS). (Created and modified from free online Google maps by using Microsoft office 365 power point).

Water and sediment samples were collected on the 1st and 2nd of June 2023 from three different sites on the Red Sea coast of Egypt as shown in Fig. 1. The first and second sites are chosen as a chemical pollution gradient source in the Red Sea, while the third one was chosen far from the chemical pollution source. The first site, El-Hamraween (HMR) (N: 26° 15’ 4.622’’, E: 34° 12’ 11.253’’), is a berth close to the oldest and largest phosphate harbor on the Egyptian Red Sea coast, located in the central part of the Red Sea coast, about 60 km south of the port of Safaga. This site was chosen as a source of chemical pollution in the Red Sea. The second site, Abo El-Swater (SWT) (N: 26° 12’ 19.153’’, E: 34° 13’ 9.94’’), which is a sharm (surrounded by corals), is near the first site; around 5 kilometers south of El-Hamraween and 14 kilometers north of Quseir. This site is characterized by its rich biodiversity and has the most popular night dive and multiple diving camps that facilitate underwater exploration and research. The third site is Om El-Abas (ABS) (N: 24° 31’ 28.536’’, E: 35° 8’ 14.144’’), located within the Wadi El-Gemal National Park, recognized for its ecological importance as a natural drainage outlet for floodwaters and about 21 km away from Mangrove forest. Sampling of three replicates of sediment and water were collected from two zones from each site. First zone represents the low intertidal habitat (L-Int) which faces seawater, while the second zone is high intertidal habitat (H-Int) which faces the seashore. Water samples (1 L) were kept in bottles in which each sample was separated into two parts, drops of nitric acid were added to one for Cd measurement and preserved in fridge until analysis. The other bottle was kept frozen in icebox filled with ice until reached the laboratory for caffeine determination. Sediment samples were separated into two parts, first part (0.5 kg) kept in plastic cases and preserved in icebox with ice for measuring caffeine concentration. The second part (0.5 kg) was dried and transported to the Lab for measuring grains size, organic matter, carbon, and concentration of cadmium.

### Physicochemical variables

During sampling, water temperature (W_Temp, °C) and dissolved oxygen (DO, mg/L) were determined using a portable water quality meter (MIC, Model 98725, Taiwan). The total dissolved solids (TDS, mg/L) was measured using a digital TDS handheld meter (hold, Guangdong, China). The Eutech instrument (EcoScan pH 6, Thermo Fisher Scientific, Germany) was used to record the water pH and electrical conductivity (Cond, µS/cm). In the laboratory, a sieving technique was used for sediment grain-size analysis according to Folk^37^. Three main groups were determined: the Fine sediment group (FSG) includes fractions < 0.250 mm, Medium sediment group (MSG) includes fractions between 0.5 mm and 1 mm, and Coarse sediment group (CSG) includes fractions > 1 mm. Organic matter (OM) and carbonate were measured by ignition method at (500℃ and 1000℃) respectively according to Heiri^38^.

Dry sediment (0.62 ± 0.06 gm) was digested by concentrated (65%) HNO_3_ in a Pyrex tube. Samples were boiled in sand bath until the samples dried then re-dissolved in 25 ml distilled water and filtrated. In Laboratory for Chemical Analysis, Faculty of Science, Assiut university, the Cd concentrations in the digestive sediment and water samples were determined using a flame atomic absorption spectrophotometer (Buck model 210 VGP) with an air-acetylene flame and hollow cathode lamp, lamp current (8 mA). The accuracy of the Cd concentration measurements was confirmed by performing triplicate analyses for each sample.

### Determination of caffeine concentration

For cold-preserved water and sediment samples, a single flow-through UV multiparameter sensor spectrophotometric detection was used to measure the concentrations of caffeine in sediment and water samples at the Multidisciplinary Research Center of Excellence, Assiut University (MIRCE)^39^. Triple measurements were used to determine the caffeine detection accuracy. The calibration range’s linearity ranged from 0.2 to 2 ppm. At 273 nm, the concentration was measured after it had reached the detection zone. The LOQ was 0.1 ppm with 99.9% confidence.

### Statistical analysis

Data summary and analysis were carried out using Excel Office 2013, IBM SPSS Statistics (version 20), and the PAST4 application. To investigate significant variations in physicochemical characteristics across the sites and zones under study, two-way analysis of variance (ANOVA) followed by Duncan’s test was performed. The distance-based two-way permutational multivariate analysis of variance (PERMANOVA) was conducted to examine the impact of all physicochemical variables on the collected samples, followed by PERMANOVA pairwise tests to illustrate the significance of differences between these samples. Pearson’s correlation was used to examine the relationship between physicochemical characteristics and Cd and caffeine levels. Stepwise multiple regression was performed to determine the most effective physicochemical variables on Cd and caffeine concentrations. After the data had been standardized, principal component analysis (PCA) and a hierarchical cluster of the mean values were performed to reveal underlying patterns and relationships of physicochemical variables, as well as the concentrations of caffeine and Cd in the investigated sites.

## Results

### Physiochemical variables

The investigated physicochemical variables differ among the study sites and zones (See Table S1). The mean W_Temp among the zones at different sites was (26.97 to 31.3 °C) at low intertidal zone in the SWT and the low intertidal zone in the HMR, respectively. The water pH differences among the samples was 8.03 in the low intertidal zone in SWT and 8.37 in the high intertidal zone in HMR. The water Cond. showed clear significant differences between samples at all investigated sites and was (63.67 to 91.0 µS/cm) at low intertidal zone in SWT and the low intertidal zone in HMR, respectively. DO also differ and was (5.40 to 8.47 mg/L) at the low intertidal zone in SWT and in the low intertidal zone in HMR, respectively. TDS was (245.33 to 314.67 mg/L) at low intertidal zone in ABS and the high intertidal zone in SWT, respectively. OM was (3.27 to 4.75%) at high intertidal zone in ABS and in the low intertidal zone in HMR, respectively. Differences in sediment carbonate was 41.97% in the high intertidal zone in ABS and 69.73% in the low intertidal zone in SWT. The coarse sediment grain (CSG) was (1.4 to 30.03%) at high intertidal in ABS and the low intertidal zone in SWT, respectively. Medium sediment grain (MSG) showed significant differences between the two zones in all investigated sites and was (26.98 to 65.20%) at low intertidal zone in ABS and in the high intertidal zone in SWT, respectively. The fine sediment grain (FSG) was 17.8% in the low intertidal zone in SWT and 70.08% in the high intertidal zone in ABS.

### Caffeine and cd concentration in sediment and water

The current findings of Cd and caffeine concentrations in the water and sediment for the sites under investigation in various zones vary (see Table S2). W_Cd concentrations varied from 0.24 (in SWT- L-Int and HMR L-Int) to 0.34 mg/L (in ABS H-Int). S_Cd levels were 0.44 µg/g in the in ABS- H-Int and 3.68 µg/g in HMR- H-Int. Levels of W_Caff varied amongst samples, ranging from 10.94 µg/L in SWT- L-Int to 14.17 µg/L in ABS’s high intertidal zone. In contrast, S_Caff levels varied from 0.27 µg/g in ABS- L-Int to 0.66 µg/g in HMR- H-Int.

**Fig. 2:**
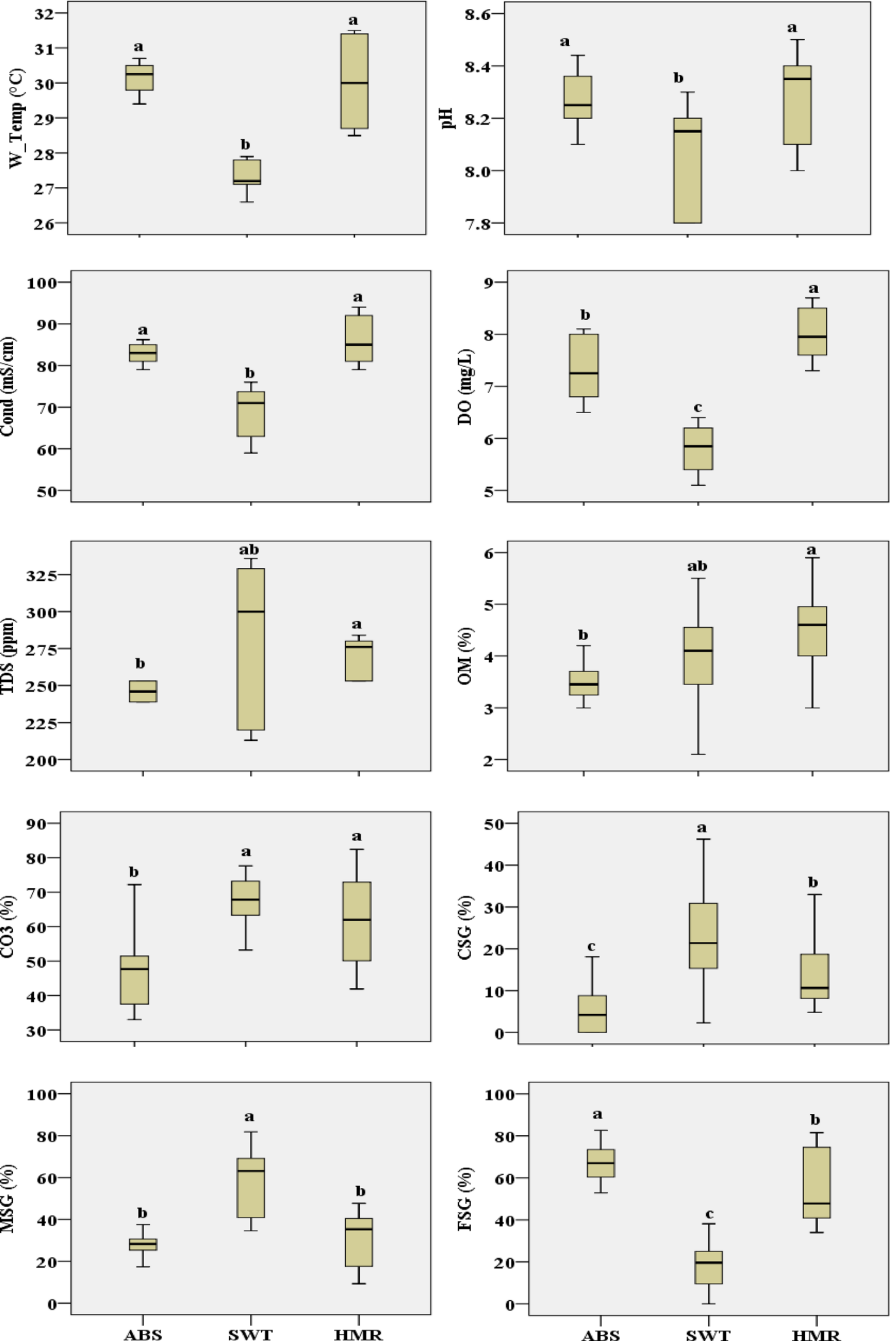
Boxplots of investigated physiochemical parameters at study sties with statistical results (similar letters for each variable show no significant difference at the 0.05 level).

**Fig. 3:**
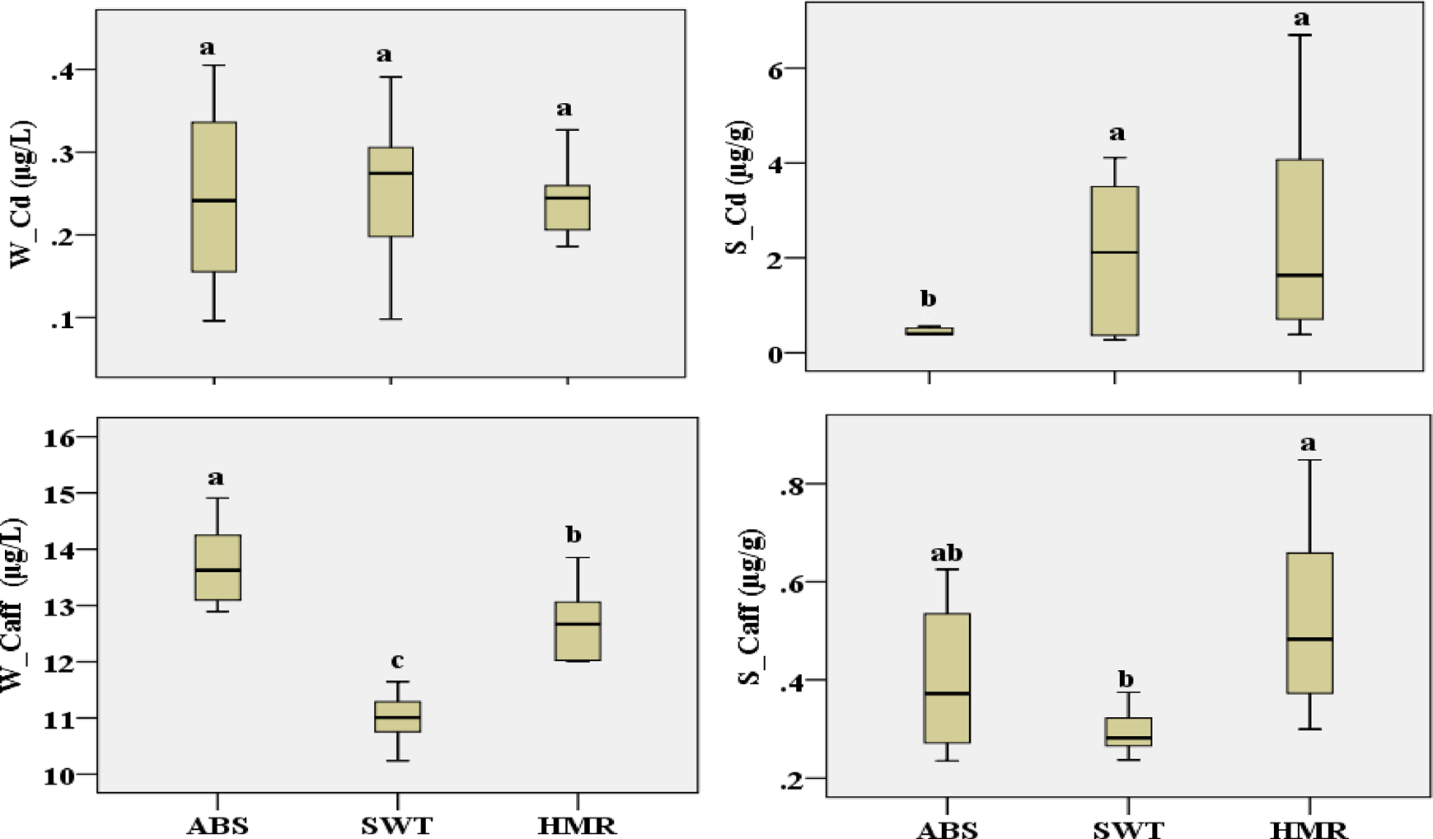
Boxplots of Cd and caffeine concentrations in water and sediment at study sites with statistical results (similar letters for each variable show no significant difference at the 0.05 level).

The statistical results showed that differences among sites were significant for all physicochemical variables investigated (*p* < 0.05). SWT site had the lowest values for W_Temp, pH, Cond., DO, and FSG as shown in Fig. 2. Except for FSG, all previous variables showed no significant differences were observed between ABS and HMR. However, ABS exhibited the lowest TDS, OM, CO3, CSG, and MSG values. On the other side HMR showed the highest values for W_Temp, pH, Cond., TDS, OM, and CO3. For water and sediment Cd and caffeine concentrations level, statistical results showed that there were significant differences among the investigated sites, except in case of water Cd. Water caffeine had the highest level of concentration in ABS, followed by HMR and then SWT. However, both Cd and caffeine in sediment had a significant high concentration levels at HMR as shown in Fig. 3.

Regarding the differences between zones, there are significant differences, In case of W_Temp, DO, CO3, and CSG, the low intertidal zone was higher than the high intertidal zone. While high intertidal zone was higher than low intertidal zone in TDS, Cd and caffeine in water and sediment (See Table 1).

**Table 1:** Mean (M) ± standard deviation (SD) of physiochemical variables and cd and caffeine concentrations for different investigated zones (H-Int: high intertidal, L-Int: low intertidal) with statistical results (W_Temp: water temperature, DO: dissolved oxygen, TDS: total dissolved solids, pH: water pH, cond: electrical conductivity, FSG: fine sediment group, MSG: medium sediment group, CSG: coarse sediment group, OM: organic matter (OM) CO_3_: carbonate content, W_Cd: water cadmium, S_Cd: sediment cadmium, W_Caff: water caffeine, S_Caff: sediment caffeine).

Habitat	H-Int	L-Int
	M ± SD	M ± SD
W_Temp (°C)	28.72 ± 0.96	29.59 ± 1.95*
pH	8.23 ± 0.18	8.18 ± 0.21
Cond (mS/cm)	78.63 ± 3.51	79.91 ± 12.46
DO (mg/L)	6.83 ± 0.59	7.24 ± 1.39*
TDS (mg/L)	277.78 ± 33.74	255.11 ± 31.95**
OM (%)	3.85 ± 0.85	4.14 ± 0.75
CO_3_ (%)	54.69 ± 15.45	62.66 ± 11.76*
CSG (%)	11.36 ± 10.48	16.73 ± 12.24*
MSG (%)	40.61 ± 19.87	37.66 ± 17.75
FSG (%)	48.04 ± 25.70	45.61 ± 23.62
W_Cd (µg/L)	0.28 ± 0.08	0.21 ± 0.07**
S_Cd (µg/g)	2.41 ± 2.22	0.87 ± 0.88**
W_Caff (µg/L)	12.78 ± 1.44	12.14 ± 1.06*
S_Caff (µg/g)	0.50 ± 0.18	0.31 ± 0.07**

*Difference is significant at the 0.05 level.

**Difference is significant at the 0.01 le vel.

### The magnitude of variability among the collected samples

Two-way PERMANOVA for all investigated physicochemical variables and caffeine and Cd concentrations in water and sediment of the investigated sites showed significant differences among sites (F = 15.491, *p* = 0.0001), zones (F = 4.653, *p* = 0.0164), and interactions (F = 3.9569, *p* = 0.007) (See Table S3). Pairwise tests showed that all sites differed significantly from each other. (See Table S4).

**Fig. 4:**
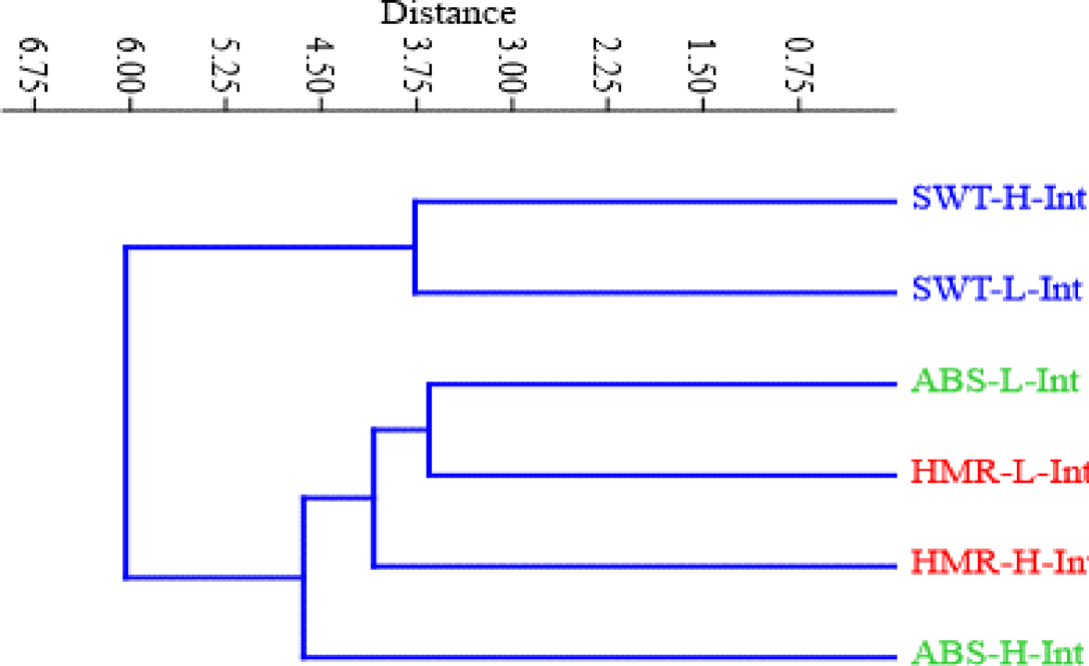
Dendrogram shows the collected samples classification at the investigated sites based on the physiochemical variables and Cd and caffeine concentrations in water and sediment (after standardizing the collected data).

At a Euclidean distance of 4.5, the results categorized the collected samples into three clusters. The first cluster consisted of SWT samples of the high intertidal and the low intertidal zones. The second cluster included the low intertidal zone of ABS and both the high intertidal and the low intertidal zones of HMR, while the high intertidal zone of ABS was separated from the other samples in the third cluster as shown in Fig. 4.

### Effect of physicochemical variables on cd and caffeine concentrations

The correlations between the studied physicochemical variables and Cd and caffeine concentrations in water and sediment are illustrated in Table 2. These partial correlations illustrate that W_Cd had a negative correlation with CSG (r = −-0.351). S_Cd had a strong positive correlation with TDS (*r* = 0.337), whereas W_Caff had a strong positive correlation with W_Temp, pH, Cond., Do, and FSG (*r* = 0.596, *r* = 0.454, *r* = 0.504, *r* = 0.507, *r* = 0.743), respectively, and a negative correlation with TDS, carbonate, CSG, MSG (*r*=-0.359, *r*= -0.642, *r*= -0.587, *r*= -0.607 respectively). S_Caff showed a positive correlation with FSG, W_Cd, and W_Caff (*r* = 0.357, *r* = 0.414 and *r* = 0.459 respectively) and a negative correlation with MSG (*r*= -0.348).

Stepwise multiple regression was applied to select the most effective variables with a significant impact on cadmium and caffeine. Table 3 illustrate these variables and the equations show their relationships with Cd and caffeine concentrations. The results indicate that caffeine concentrations in water are correlated and mainly affected by FSG, DO, and S_Caff and are negatively correlated with organic matter. While caffeine concentration in the sediment was mainly positively affected by W_Caff and W_Cd. On the other hand, the cadmium concentration in water was affected by S_Caff and negatively correlated with DO and CSG. Whereas cadmium concentration in the sediment was mainly affected by TDS (See Table 2).

**Table 2:** Pearson correlation coefficients (r) between the investigated physiochemical variables and cd and caffeine concentration in study sites.

	W_Temp	pH	Cond	DO	TDS	OM	CO3	CSG	MSG	FSG	W_Cd	S_Cd	W_Caff
pH	0.421*												
Cond	0.889**	0.298											
DO	0.880**	0.467**	0.901**										
TDS	-0.236	-0.127	-0.053	-0.104									
OM	0.11	0.258	0.18	0.29	-0.022								
CO3	-0.277	-0.298	-0.158	-0.119	0.252	0.531**							
CSG	-0.592**	-0.347*	-0.500**	-0.403*	-0.092	0.29	0.578**						
MSG	-0.578**	-0.490**	-0.502**	-0.492**	0.706**	-0.068	0.488**	0.261					
FSG	0.723**	0.540**	0.621**	0.568**	-0.497**	-0.085	-0.648**	-0.674**	-0.889**				
W_Cd	-0.107	-0.066	-0.145	-0.206	0.087	-0.214	-0.200	-0.351*	0.095	0.094			
S_Cd	-0.246	0.123	-0.052	0.018	0.337*	0.042	0.1	0.133	0.106	-0.144	0.048		
W_Caff	0.596**	0.454**	0.504**	0.507**	-0.359*	-0.312	-0.642**	-0.587**	-0.607**	0.743**	0.226	-0.073	
S_Caff	0.109	0.286	0.223	0.221	-0.016	0.009	-0.318	-0.190	-0.348*	0.357*	0.414*	0.06	0.459**

*Correlation is significant at the 0.05 level.

**Correlation is significant at the 0.01 level.

**Fig. 5:**
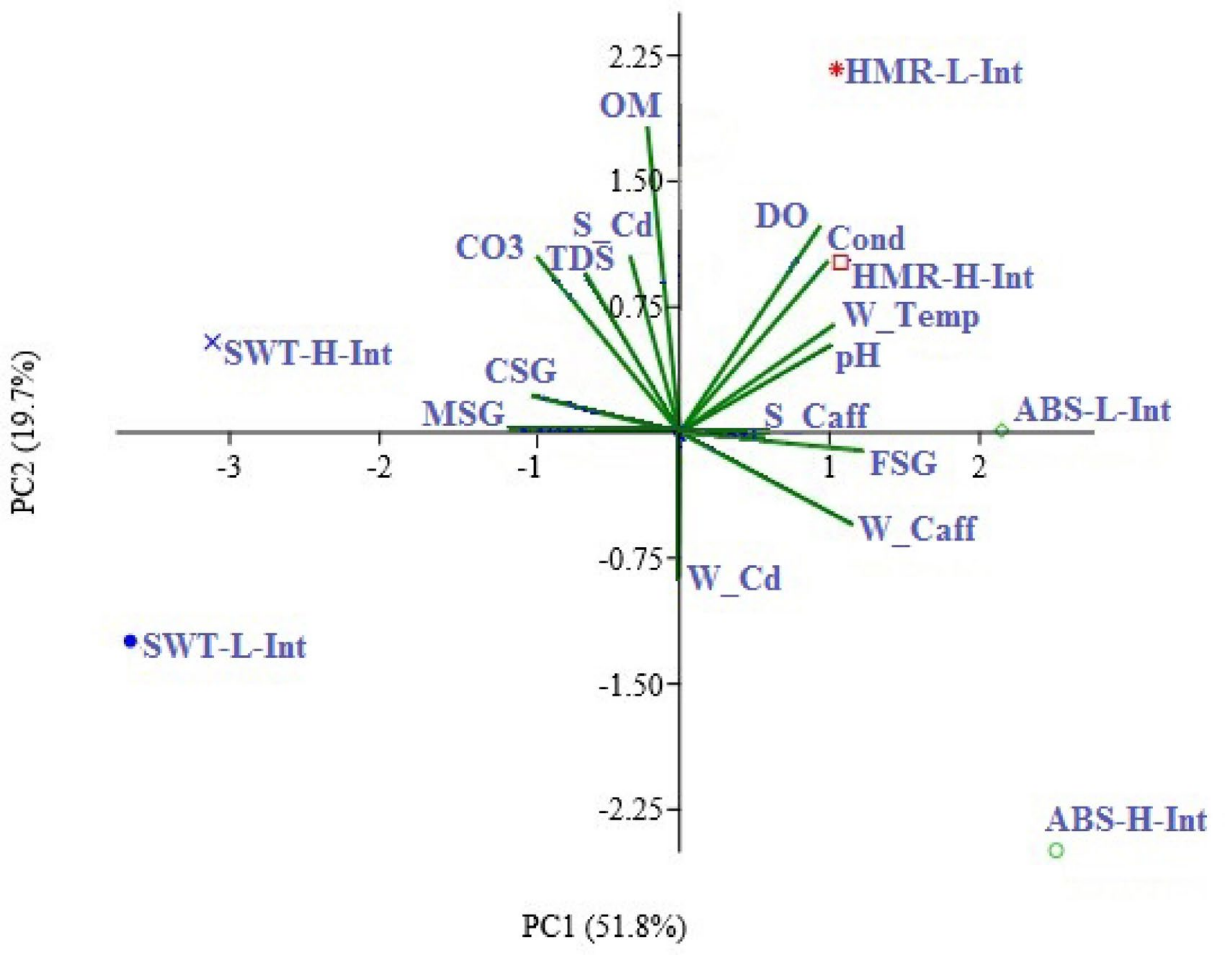
Principal component analysis (PCA) for the physiochemical variables and Cd and caffeine concentrations in water and sediment at study sites (after standardizing the collected data). Notation of variables: W_Temp: water temperature, DO: dissolved oxygen, TDS: total dissolved solids, pH: water pH, Cond: electrical conductivity, FSG: Fine sediment group, MSG: Medium sediment group, CSG: Coarse sediment group, OM: Organic matter (OM) CO3: carbonate content, W_Cd: water cadmium, S_Cd: sediment cadmium, W_Caff: water caffeine, S_Caff: sediment caffeine, ABS-H-Int: Om El-Abas High intertidal, ABS-L-Int: Om El-Abas low intertidal, SWT-H-Int: Abo El-Swater High intertidal, SWT-L-Int: Abo El-Swater low intertidal, HMR-H-Int: El-Hamraween High intertidal, HMR-L-Int: El-Hamraween low intertidal.

Principal component analysis (PCA) revealed a direct correlation between W_Cd and W_Caff, S_Caff, Cond, FSG, PH, W_Temp, and DO. However, a negative correlation was observed between W_Cd and S_Cd, OM, TDS, CO3, CSG, and MSG as shown in Fig. 5.

According to Principal component analysis (PCA), PC1 elucidated 51.8% of total fluctuations of physicochemical variables and Cd and caffeine concentrations, separating both zones of ABS and HMR sites in the positive side from SWT zones. However, PC 2 elucidated 19.7% separated high intertidal zone in SWT and HMR zones in the positive site. PCA showed a direct correlation between W_Cd and W_Caff, S_Caff, Cond, FSG, PH, W_Temp, and DO. However, they are negatively correlated with S_Cd, OM, TDS, CO3, CS, and MSG as shown in Fig. 5.

## Discussion

Despite the great interest in pharmaceutical residues, especially caffeine, research on this topic is very limited in Africa, where only three studies have been published at the continental level to detect caffeine concentration^8,9,40^. In Egypt, Tawfik et al.^9^ concerned with the occurrence of caffeine in the Nile River. This study is the first to focus on caffeine concentration levels on the Red Sea coast of Egypt as a marine environment. Ali et al.^14^ detected thirteen PPCPs on the Red Sea coast of Saudi Arabia. They identified caffeine as one of the most abundant PPCPs, with concentrations in the range of 62 to 7708 ng/L. The current study highlighted the importance of monitoring pharmaceutical residues in the water of the Red Sea to follow its quality.

The current study demonstrated the presence of caffeine at varying concentrations in water and sediment samples at the investigated sites. The caffeine concentrations of the water samples were in the range of 10.94 to 14.17 µg/L. These values are relatively higher than the previously measured concentration in the Red Sea in Saudi Arabia (7708 ng/L)^14^. This difference may be related to the fact that Egypt is relatively more highly populated than Saudi Arabia and more activities close to the sea coast. Another reason, the caffeine level may have been duplicated over these eight years in the Red Sea. The fluctuation of caffeine concentration due to the time factor and caffeine dispersion in the aquatic environment, which is related to its high consumption coupled with its relative stability under environmental conditions. Caffeine is relatively stable under different conditions, including seawater, and has high water solubility (approximately 13.0 g/L), low octanol-water coefficient (log kow = − 0.07), low volatility^41–43^, and a reported half-life of 100–240 days^22^.

Considering the various levels of caffeine in marine ecosystems, The Red Sea of Egypt has a relatively high level of caffeine. Recent estimations of caffeine concentrations within European marine environments have indicated levels of 4.9 to 677 ng/L in the North Sea (including Germany, Holland, Norway, and Sweden), 4 to 804 ng/L in the Atlantic Ocean (encompassing France and Portugal), 4.5 to 3390 ng/L in the Mediterranean Sea (including Greece and Turkey), Taiwan (16.92 ng/ L)^44^, and 8.2 to 1110 ng/L in the Adriatic Sea (Italy)^45^. Furthermore, notably elevated caffeine concentrations have been detected along the coasts of the United States (5860 ng/L)^46^, Japan (8230 ng/L)^47^, Australia (11,000 ng/L)^48^, and Spain (18,493 ng/ L)^45^. These ratios make the Red Sea of Egypt in the third rank after Spain (18,493 ng/ L) and Taiwan (16.92 ng/ L).

It is worth noting that the high caffeine concentration in Spain was related to the site of sample collection, which was the effluent of the Noia WWTP, which is expected to have high concentration of caffeine. While high level of caffeine in Egypt may be related to the geographic boundaries of the Red Sea, as it is a semi-closed sea and the nature of each site and the impact of anthropogenic effects on it, in addition to the high population density and the rise in tourism and recreational activities during the sea-bathing season has been associated with a substantial increase in caffeine levels in coastal waters^49^. Rangel-Buitrago et al.^50^ demonstrate that increased population density and tourism lead to urban expansion and higher wastewater loads, intensifying environmental pressures on coastal areas. Future research should specifically focus on correlating caffeine levels with demographic, environmental, and tourism data to provide a stronger evidence base.

On the other hand, the current study demonstrated the accumulation of caffeine in coastal sediments, and the caffeine concentrations of the sediment samples ranged from 0.27 to 0.66 µg/g. Caffeine concentrations in coastal sediments have been documented in a few studies, with values ranging from 1.90 to 12.20 ng/g in Spain^51^ and from 0.31 to 23.4 ng/g in Brazil^52^.The highest concentration of caffeine found in the Red Sea sediment may be related to the naturalness of the semi-closed system of this Sea, as mentioned before. The Red Sea is also stressed by many anthropogenic activities, as it is a shipping corridor that connects the world East and the West.

The other investigated microcontaminate is cadmium, which also originates from anthropogenic activities. Generally, the Cd concentration in marine environments reflects the anthropogenic inputs of Cd-containing materials^53^. The present study recorded cadmium concentrations in water and sediment samples ranged between (0.15 to 0.34 µg/L and 0.44 to 3.68 µg/g, respectively). Abouhend and El-Moselhy^54^ indicated that the annual means of cadmium concentrations in seawater of the Red Sea (0.22 to 0.39 µg/l), while in sediment the concentrations ranged between 2.03 and 3.86 µg/g. El-Sokkary (2022) detected Cd in the Red Sea sediment 0.14 to 1.07 µg/g. The concentrations detected in this study ensure that these ratios are higher than the permissible sediment quality guidelines levels mentioned by^54,55^.

The present study demonstrated significant variations in water and sediment caffeine and Cd concentrations and investigated physicochemical variables among the study sites. According to these differences, the PREMANOVA test illustrated that the three sites are significantly different from each other (See Tables S3, S4), which is mainly related to the fact that these sites are under different anthropogenic impacts. Hawash et al.^8^ showed that anthropogenic pollution is the source of caffeine. They illustrated that caffeine is thought to primarily originate from human activity in restaurants, public spaces, and residential homes. In contemporary sewage treatment plants (STPs), however, caffeine is typically easily retained and represents the local consumption profile of beverages containing caffeine, which are then expelled and subjected to local sewage treatment processes before being discharged into the environment. As a result of these anthropogenic activities, caffeine is today considered as a chemical indicator of anthropogenic influence on aqueous environments and clearly demonstrate here also the polluting effect of the nearby community on the examined marine coastal environments^14^.

Although ABS is present in protected area, it was associated with a relatively high concentration of caffeine in water. This may be related to the natural source of caffeine, where the presence of mangrove and sea grass is close to this site. Previous studies indicate that Caffeine is found in surface water from natural sources, as it can be found in sea grass and algae, and it is detected in macroalgae samples with a maximum concentration of 41.3 ng/g (on a dry weight basis = dw)^10,56^. Another reason, this site is considered a natural drainage outlet for floodwaters, which carry micropollutants from the terrestrial environment, including caffeine. On the other side, caffeine concentration in the sediments was relatively high in HMR. Numerous factors, including the impact of terrigenous inputs from Wadi Al-Hamraween, which faces the study area, contributed to the increased caffeine content in the sediments at this location. Another factor is the relatively higher metal content of sediments in this site, which led a previous study to suggest Al-Hamraween is a contaminated area^27,29,30^. Tawfik et al.^9^ reported a positive correlation between Zn and caffeine concentrations in sediment in the aquatic environment. A similar positive association between Cd and caffeine was observed in the current study. These studies collectively suggest that the presence of caffeine in aquatic sediments is often associated with elevated levels of certain heavy metals, particularly Zn and Cd, and that environmental conditions can influence this relationship. Another important factor may increase the caffeine concentration in the sediment of HMR, that this site is characterized by a relatively high OM and fine sediment grains FSG, which helps in clutching heavy metal and caffeine^57,58^. Bayen et al.^57^ illustrated that there is a significant positive linear correlation between the levels of caffeine in sediments and sediment organic matter.

Additionally, due to the presence of high levels of phosphate dust from the closest phosphate harbor, the current results showed a high accumulation of Cd in the sediment of HMR and the closest site SWT. Madkour et al.^30^ illustrated that the considerably high Cd at Al-Hamraween was mostly due to phosphate raw materials that were naturally enriched by Cd and in the other megacities to landfilling and coastal based activities. Thick phosphate dust layer covers the original sediments of Aqaba harbor and the freshly deposited raw phosphate powder contains high levels of heavy metals^59^. The nature of the HMR sediment, which is fine grains, also plays a role in the clutching of cadmium. The fine fraction sediments (< 0.125mmand < 0.063 mm) are considerably effective in the contaminants accumulation and transfer to the marine ecosystem^58^.

The present results clearly indicate significant differences between the study zones in Cd and caffeine concentrations and investigated physicochemical variables (See Table 1). The high intertidal zone, which faces the sea shore, showed higher Cd and caffeine accumulation than the low intertidal zone. This may be because this location is receiving contamination from the terrestrial anthropogenic impacts from the terrestrial are mainly more from the sea. In addition, wave action transports pollutants from the sea and settle them on the shore. Madkour and Dar^58^ illustrated that the accumulation of contaminants recorded their higher contents in sediments at and near the beach and gradually decreased seaward. This can explain why the high intertidal zone of ABS was separated in a single group according to cluster analysis as shown in Fig. (4), which is characterized by a relatively high concentration of caffeine in sediment. Also, the results of cluster analysis separated the low intertidal zone of ABS and both low and high intertidal of HMR in a single group as shown in Fig. (4). This may relate to the nature of the sediment in HMR and ABS, where both of them are characterized by fine grain size and relatively high OM content. This, as mentioned previously, plays a role in the accumulation of cadmium and caffeine, which they clutch with fine particles and make it higher in density to be held in water, which makes it settle in sediment.

The current partial correlation analyses showed that caffeine concentrations in seawater were highly associated with the studied physicochemical variables. Water caffeine was strongly positively correlated with W_Temp, pH, Cond., Do, and FSG and negatively correlated with TDS, carbonate, CSG, and MSG (See Table 2). Previous studies have proven that the bioavailability of micropollutants like caffeine is tightly dependent on environmental conditions^9,60–62^. Puckowski et al.^62^ illustrated that when pharmaceutical residues enter the environment, their fate and effects are determined by many factors, including their properties and physicochemical characteristics of the environment.

Stepwise multiple regression selected caffeine and cadmium concentrations in water as strong effective factors for the accumulation of caffeine in sediment (See Table 3). Tawfik et al.^9^ found a similar correlation between caffeine accumulation and heavy metal (Zn) concentrations in aquatic environments. This synergistic relationship between caffeine and heavy metals highlights a critical environmental concern associated with the co-occurrence of heavy metals and pharmaceutical contaminants, raising concerns regarding their bioavailability and their effect on both the environment and human health. This highlights the necessity for further investigation into these interactions in future studies. On the other hand, the present results indicate that the accumulation of Cd in the sediment is strongly affected by TDS. This can be explained by the role of cation exchange; however, the increase in TDS, which contains divalent cations (e.g., Ca and Mg), improved the binding of Cd ions to sediment particles. Vega et al.^63^ illustrated that the sorption of heavy metal ions (Cd, Cu and Pb) by soil horizons is mainly a cation exchange process, with Mg^2+^ replacing the majority of the replaced cations, followed by Ca^2+^, Al^3+^ and K^+^.

**Table 3:** Stepwise multiple regression between cd and caffeine concentrations in water and sediment with the investigated physiochemical variables.

Dependent Variable	Selected variable	*p*-value	*R* ^2^	Std. error of the Estimate	Model F-value (*p*-value)	Regression equations
W_Caf	Constant	< 0.001	0.71	0.73	19.24 (< 0.001)	W_caf = 10.37 + 0.46FSG − 0.36OM + 0.3DO + 0.23S_Caf
	FSG	0.001				
	OM	0.002				
	DO	0.026				
	S_Caff	0.031				
SCaf	Constant	0.117	0.31	0.14	7.489 (0.002)	S_caf = -3.77 + 0.39 W_Caf + 0.33W_Cd
	W_caf	0.014				
	W_Cd	0.035				
W_Cd	Constant	< 0.001	0.44	0.64	8.49 (> 0.001)	W_Cd = 0.47 + 0.43 S_Caf-0.49 DO-0.47 CSG
	S_Caff	0.003				
	DO	0.002				
	CSG	0.003				
S_Cd	Constant	0.181	0.11	1.76	4.36 (0.44)	S_Cd = -3.17 + 0.34 TDS
	TDS	0.044				

Based on PC axis (1) (51.8%), The present PCA result revealed that the different interactions between the investigated micropollutant (caffeine and cadmium) and physicochemical variables separated SWT from HMR and ABS. HMR and ABS were directly correlated with W_Cd and W_Caff, S_Caff, Cond, FSG, PH, W_Temp, and DO. However, they were negatively correlated with S_Cd, OM, TDS, CO3, CSG, and MSG as shown in Fig. (5).This demonstrates how site-specific physicochemical factors significantly affect the distribution and behavior of pollutants (such as caffeine and cadmium) in seawater and coastal sediments. Recent research across diverse locations has found that pollution, encompassing organic pollutants and heavy metals, in seawater and coastal deposits is strongly site-specific, leading to considerable variations across different areas^26,64–66^. Together, these studies show that there is substantial spatial variability in the pollutants found in coastal environments rather than their uniformity. The main causes of this variability are anthropogenic influences and physicochemical factors. It is essential to comprehend these site-specific elements in order to manage pollution and protect the environment in coastal areas.

## Conclusion

The current results indicate a high concentration of caffeine in the Red Sea due to various influencing factors including anthropogenic impacts. The level of caffeine accumulation is expected to increase over time in conjunction with an increase in human activity. Differences in anthropogenic impacts can affect the levels of caffeine and cadmium in the environment, as evidenced by the varying accumulation levels in different investigated sites. Nevertheless, the correlation between caffeine, cadmium, and various anthropogenic activities and environmental factors remains unclear and requires extensive research. Caffeine and cadmium concentrations were shown to be positively correlated with Cond, FSG, PH, W_Temp, and DO. W_Cd was shown to be negatively correlated with S_Cd, OM, TDS, CO3, CSG, and MSG. The results revealed a synergistic interaction between caffeine and cadmium. As a result, it is critical to investigate heavy metals and their relationship with caffeine, as this is an environmental concern that endangers ecosystems and human health. Effective ways to reduce the different human activities that have a detrimental influence on the general health of the ecosystem should also be pursued. Future study should focus on examining these relationships.

## Supplementary Information

Below is the link to the electronic supplementary material.

Supplementary Material 1

## Data Availability

The datasets generated during and/or analysed during the current study are available from the corresponding author on reasonable request.
