# Supplementary material for: Occurrence and concentration of caffeine and cadmium as micropollutants in the Red Sea coast, Egypt
Source: Sci Rep. 2026 Feb 23;16:7476. doi: 10.1038/s41598-026-38344-7 (PMC12929714; doi:10.1038/s41598-026-38344-7)
Supplement: Supplementary file 1 — Supplementary Material 1 [file 41598_2026_38344_MOESM1_ESM.docx]

**Occurrence and concentration of caffeine and cadmium as micropollutants in the Red Sea coast, Egypt**

Samaa G. El-Sokkary^a^, Khaleid F. Abd El-Wakeil^a*^ and Ahmad H. Obuid-Allah^a^

#### ^a^ *Zoology and Entomology Department, Faculty of Science, Assiut University, Egypt*

*^*^ Corresponding author:* [kfwakeil@yahoo.com](mailto:kfwakeil@yahoo.com), kfwakeil@aun.edu.eg

**Supplementary materials**

**Table S1.** Mean (M) ± standard deviation (SD) of physiochemical variables for the study sites (ABS: Om El-Abas, SWT: Abo El-Swater, HMR: El-Hamraween) and zones (H-Int: High intertidal, L-Int: low intertidal).

| **Site** | **ABS** | | | | | | **SWT** | | | | | | **HMR** | | | | | |
| --- | --- | --- | --- | --- | --- | --- | --- | --- | --- | --- | --- | --- | --- | --- | --- | --- | --- | --- |
|  | **H-Int** | | | **L-Int** | | | **H-Int** | | | **L-Int** | | | **H-Int** | | | **L-Int** | | |
|  | M | ± | SD | M | ± | SD | M | ± | SD | M | ± | SD | M | ± | SD | M | ± | SD |
| **W_Temp (°C)** | 29.80 | ± | 0.36 | 30.50 | ± | 0.18 | 27.63 | ± | 0.34 | 26.97 | ± | 0.29 | 28.73 | ± | 0.23 | 31.30 | ± | 0.24 |
| **pH** | 8.22 | ± | 0.12 | 8.31 | ± | 0.11 | 8.10 | ± | 0.24 | 8.03 | ± | 0.19 | 8.37 | ± | 0.05 | 8.20 | ± | 0.24 |
| **Cond (μS/cm)** | 80.67 | ± | 1.37 | 85.07 | ± | 0.99 | 74.23 | ± | 1.40 | 63.67 | ± | 4.50 | 81.00 | ± | 1.79 | 91.00 | ± | 3.22 |
| **DO (mg/L)** | 6.77 | ± | 0.23 | 7.87 | ± | 0.29 | 6.20 | ± | 0.18 | 5.40 | ± | 0.27 | 7.53 | ± | 0.19 | 8.47 | ± | 0.23 |
| **TDS (mg/L)** | 246.67 | ± | 6.35 | 245.33 | ± | 6.35 | 314.67 | ± | 27.80 | 251.33 | ± | 54.05 | 272.00 | ± | 14.89 | 268.67 | ± | 12.53 |
| **OM (%)** | 3.27 | ± | 0.18 | 3.75 | ± | 0.31 | 4.03 | ± | 1.01 | 3.93 | ± | 0.85 | 4.25 | ± | 0.87 | 4.75 | ± | 0.64 |
| **CO_3_ (%)** | 41.97 | ± | 15.00 | 50.50 | ± | 2.40 | 66.08 | ± | 7.35 | 69.73 | ± | 6.38 | 56.03 | ± | 13.41 | 67.75 | ± | 12.45 |
| **CSG (%)** | 1.40 | ± | 2.17 | 9.28 | ± | 5.28 | 15.25 | ± | 8.55 | 30.03 | ± | 11.62 | 17.42 | ± | 10.69 | 10.88 | ± | 5.17 |
| **MSG (%)** | 28.52 | ± | 7.03 | 26.98 | ± | 3.48 | 65.20 | ± | 4.89 | 52.18 | ± | 21.76 | 28.10 | ± | 13.42 | 33.82 | ± | 13.36 |
| **FSG (%)** | 70.08 | ± | 8.79 | 63.73 | ± | 7.95 | 19.52 | ± | 10.58 | 17.80 | ± | 13.04 | 54.52 | ± | 21.12 | 55.30 | ± | 15.13 |

W_Temp: water temperature, pH: water pH, Cond: conductivity, DO: dissolved oxygen, TDS: total dissolved solids, OM: organic matter, CO_3_: carbonate, CSG: coarse sediment grains, MSG: medium sediment grains, FSG: fine sediment grains

**Table S2.** Mean (M) ± standard deviation (SD) of Cd and caffeine concentrations in water and sediment for the study sites (ABS: Om El-Abas, SWT: Abo El-Swater, HMR: El-Hamraween) and zones (H-Int: High intertidal, L-Int: low intertidal).

| **Site** | **ABS** | | | | | | **SWT** | | | | | | **HMR** | | | | | |
| --- | --- | --- | --- | --- | --- | --- | --- | --- | --- | --- | --- | --- | --- | --- | --- | --- | --- | --- |
|  | **H-Int** | | | **L-Int** | | | **H-Int** | | | **L-Int** | | | **H-Int** | | | **L-Int** | | |
|  | M | ± | SD | M | ± | SD | M | ± | SD | M | ± | SD | M | ± | SD | M | ± | SD |
| **W_Cd  (μg/L)** | 0.34 | ± | 0.04 | 0.15 | ± | 0.04 | 0.26 | ± | 0.10 | 0.24 | ± | 0.08 | 0.25 | ± | 0.04 | 0.24 | ± | 0.05 |
| **S_Cd (µg/g)** | 0.44 | ± | 0.07 | 0.50 | ± | 0.17 | 3.10 | ± | 1.05 | 0.80 | ± | 1.08 | 3.68 | ± | 2.92 | 1.32 | ± | 1.01 |
| **W_Caff  (μg/L)** | 14.17 | ± | 0.70 | 13.28 | ± | 0.38 | 11.04 | ± | 0.47 | 10.94 | ± | 0.48 | 13.12 | ± | 0.47 | 12.21 | ± | 0.40 |
| **S_Caff (µg/g)** | 0.54 | ± | 0.08 | 0.27 | ± | 0.02 | 0.31 | ± | 0.06 | 0.28 | ± | 0.02 | 0.66 | ± | 0.16 | 0.38 | ± | 0.08 |

W_Cd: water cadmium, S_Cd: sediment cadmium, W_Caff: water caffeine, S_Caff: sediment caffeine

**Table S3.** Two-way PERMANOVA for physicochemical variables and caffeine and Cd concentrations in water and sediment at the investigated samples.

| **Source** | **Sum of sqrs** | **df** | **Mean square** | **F** | **p-value** |
| --- | --- | --- | --- | --- | --- |
| Site | 37527 | 2 | 18763 | 15.491 | **0.0001** |
| Habitat | 5635.6 | 1 | 5635.6 | 4.6527 | **0.0164** |
| Interaction | 9585.4 | 2 | 4792.7 | 3.9569 | **0.007** |
| Residual | 36337 | 30 | 1211.2 |  |  |
| Total | 89085 | 35 |  |  |  |

**Table S4.** PERMANOVA pairwise tests for differences between the investigated sites according to standardized physicochemical variables and caffeine and Cd concentrations.

| **Sites** | **ABS-SWT** | **ABS-HMR** | **SWT-HMR** |
| --- | --- | --- | --- |
| **F** | 18.16 | 10.28 | 7.28 |
| **p-value** | **0.0003** | **0.0033** | **0.0075** |
